# Supplementary material for: Hydrogen peroxide positively regulates brassinosteroid signaling through oxidation of the BRASSINAZOLE-RESISTANT1 transcription factor
Source: Nat Commun. 2018 Mar 14;9:1063. doi: 10.1038/s41467-018-03463-x (PMC5852159; doi:10.1038/s41467-018-03463-x)
Supplement: Supplementary file 1 — Supplementary Information [file 41467_2018_3463_MOESM1_ESM.pdf]

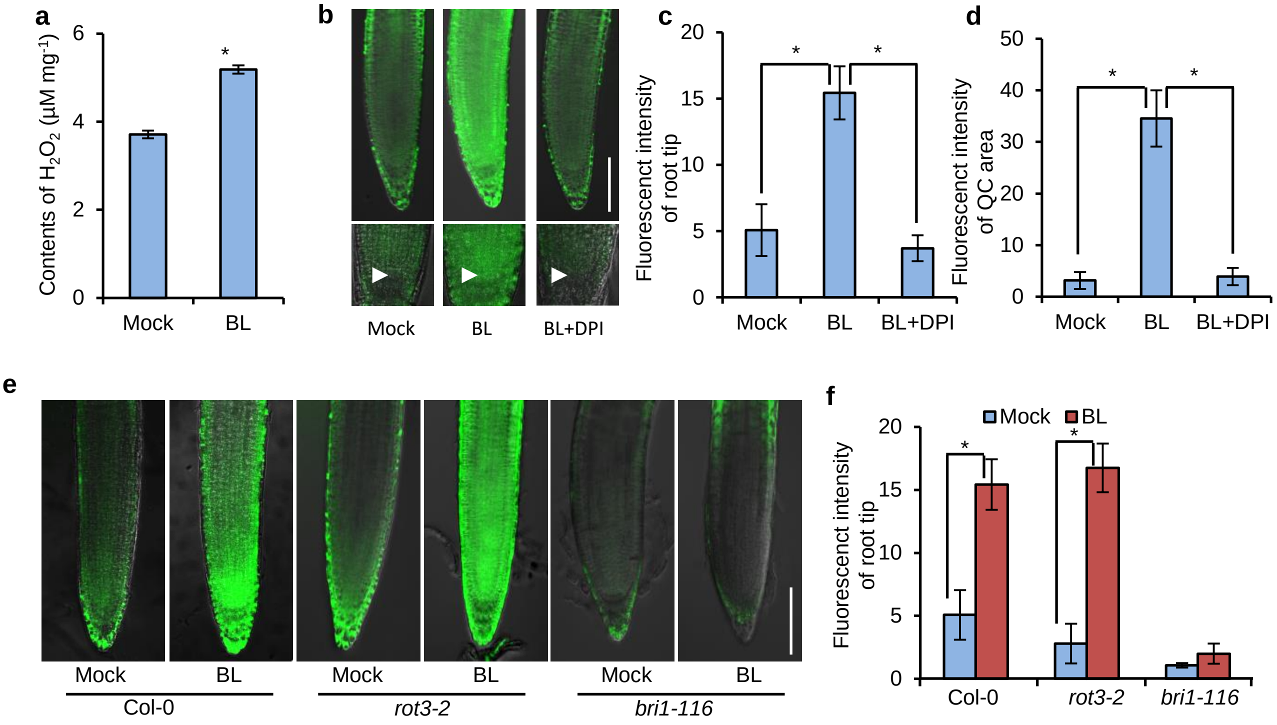

Supplementary Figure 1. BR induces the production of H_2O_2 through a BRI1-dependent pathway.

(a) H_2O_2 content was measured using the amplex red hydrogen peroxide/peroxidase assay in 5-day old light-grown Arabidopsis seedlings. Error bars represented the s.d. of three independent experiments. Asterisk indicated significant difference from mock treatment (*t* test; $*p < 0.05$). **(b)** H_2DCFDA staining for H_2O_2 in the primary root tips of Col-0 treated with BL and BL plus DPI. Lower panels showed zoom-in views of the QC area, and white arrows represented QC cells. **(c,d)** Quantification of H_2DCFDA fluorescent intensities in the root tips (c) or QC cells (d) of the seedlings in panel (b). **(e)** H_2DCFDA staining for H_2O_2 in the primary root tips of Col-0, *rot3-2*, and *bri1-116* treated with or without BL. Scale bar, 100 µm. **(f)** Quantification of H_2DCFDA fluorescent intensities in the images of panel (e). Seedlings were grown on ½ MS medium for 4 days, and then transferred to ½ MS liquid medium containing BL and/or DPI as indicated for another one day. Fluorescent signals were taken using LSM700 microscope from Zeiss. Error bars, s.d. (n=20 images). Asterisk indicated significant difference from mock treatment (t test; $*p <$ 0.05).

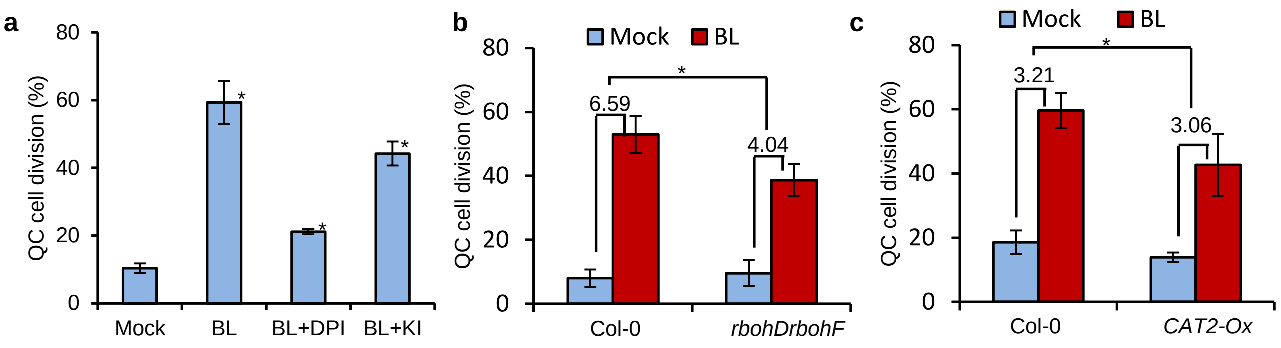

Supplementary Figure 2: The accumulation of H_2O_2 is required for BR-induced quiescent center cells division.

(a-c) Quantification of QC cell division in the root of Col-0 (a), *rbohDrbohF* (b), and *CAT2-Ox* (c) treated with or without BL and/or DPI and KI. Seedlings of Col-0, *rbohDrbohF*, and *CAT2-Ox* were grown on ½ MS medium for 4 days, and then transferred to ½ MS liquid medium with or without BL, DPI or KI for another one day. At least 50 seedlings were examined for each biological repeat. Error bars represented the s.d. of three independent experiments. *$p < 0.05$, as determined by a Student's t test.

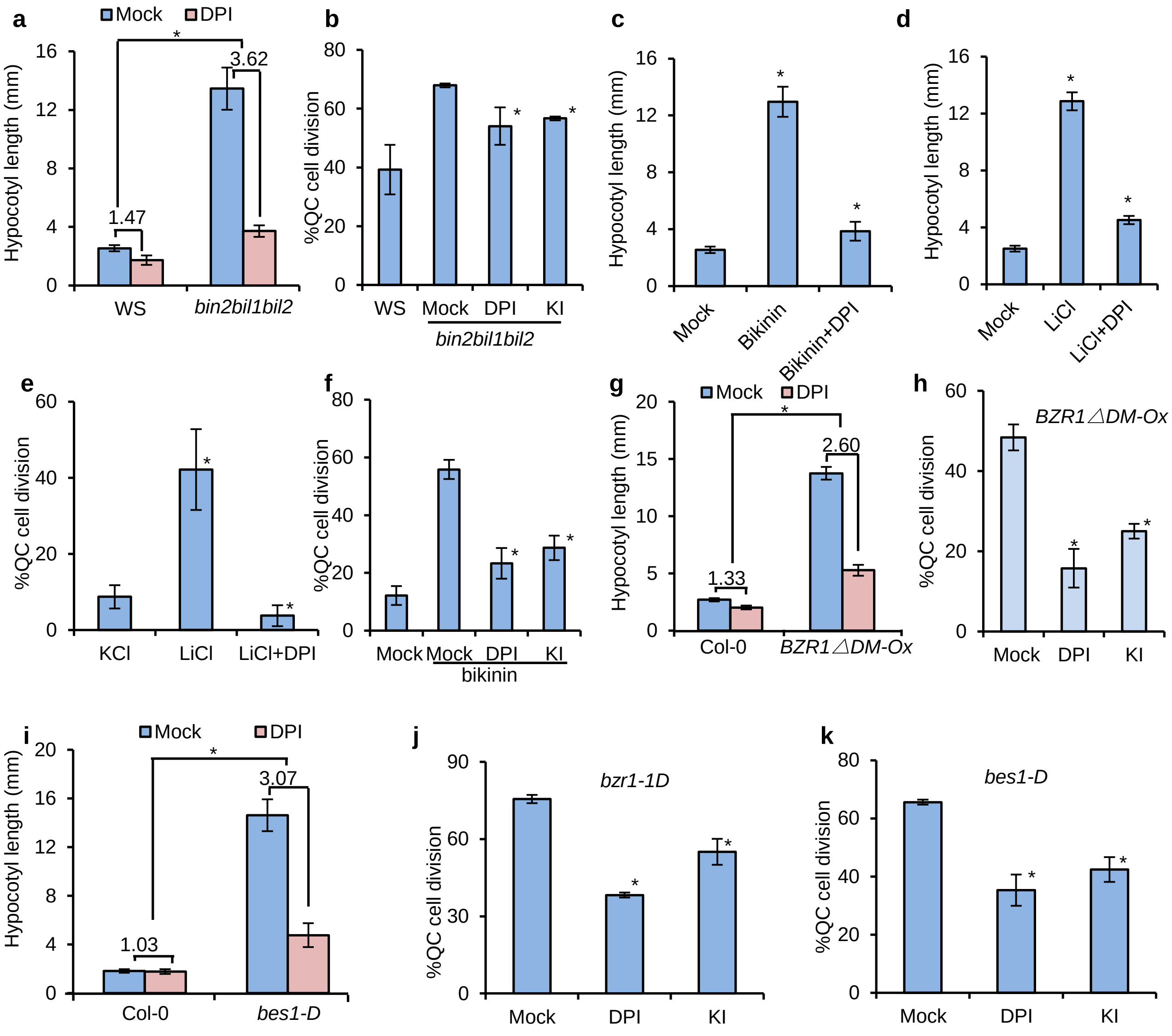

Supplementary Figure 3. H_2O_2 contributes to the BR signaling pathway by targeting the downstream components of BIN2.

(a) DPI attenuated the PPZ-resistant phenotype of *bin2bil1bil2*. **(b)** DPI and KI reduced the QC cell division in the *bin2bil1bil2* mutant. **(c,d)** DPI attenuated the bikinin and LiCl effects on the cell elongation. **(e)** DPI counteracted the function of LiCl on QC cell division. **(f)** DPI and KI reduced the bikinin-induced QC cell division. **(g)** DPI attenuated the PPZ-resistant phenotype of *BZR1△DM-Ox* transgenic plants. **(h)** DPI and KI reduced the QC cell division in *BZR1△DM-Ox* transgenic plants. **(i)** DPI attenuated the PPZ-resistant phenotype of *bes1-D*. **(j,k)** DPI and KI reduced the QC cell division in *bzr1-1D* and *bes1-D* plants. For hypocotyl elongation assays, seedlings of wild type and different mutants were grown on ½ MS medium containing different chemicals as indicated in the dark for 6 days. Error bars, s.d. (n=25 plants). *$p < 0.05$, as determined by a Student's *t* test. For QC cell division analysis, seedlings of Col-0 and different mutants were grown on ½ MS medium under constant light for 4 days, and then transferred to ½ MS liquid medium containing different chemicals as indicated for another one day. At least 50 seedlings were examined for each biological repeat. Error bars represented the s.d. of three independent experiments. Asterisk indicated significant difference from mock treatment (*t* test; *$p < 0.05$).

a

b

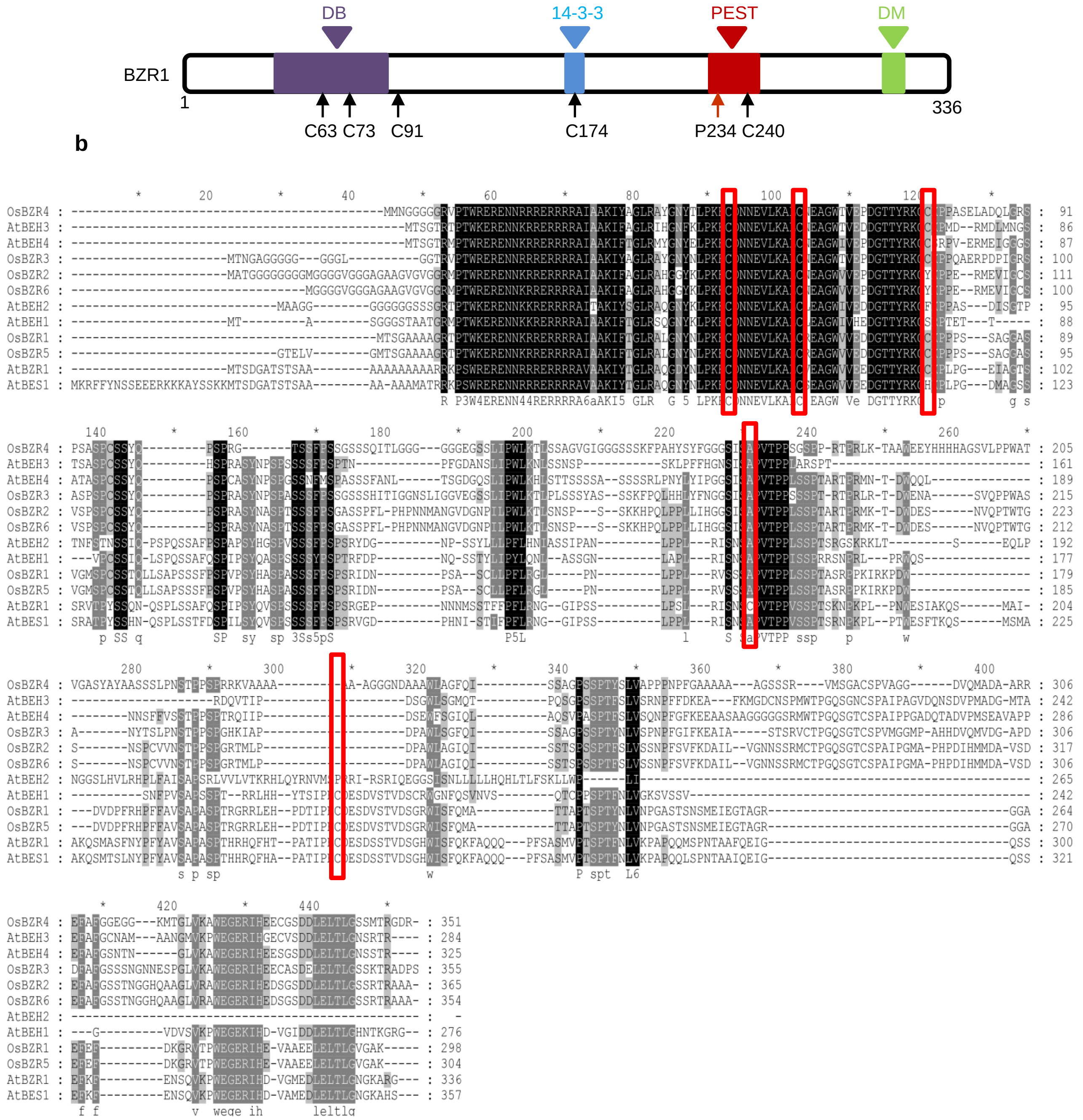

Supplementary Figure 4. Protein alignment of BZR1 family in Arabidopsis and *Oryza sativa*.

(a) Illustration depicted BZR1 protein structure. Purple box indicates DNA binding domain; dark blue indicates 14-3-3 binding domain; dark red indicates proline, glutamic acid, serine, and threonine rich (PEST) domain; light green indicates BIN2 binding domain (DM); the black arrows refer to the cysteine residues; and the red arrow refers the 234th proline. **(b)** The protein sequence alignment of BZR1 and its homologs. The alignment image was produced by CLUSTAL OMEGA software. Red box indicates five cysteine residues of BZR1.

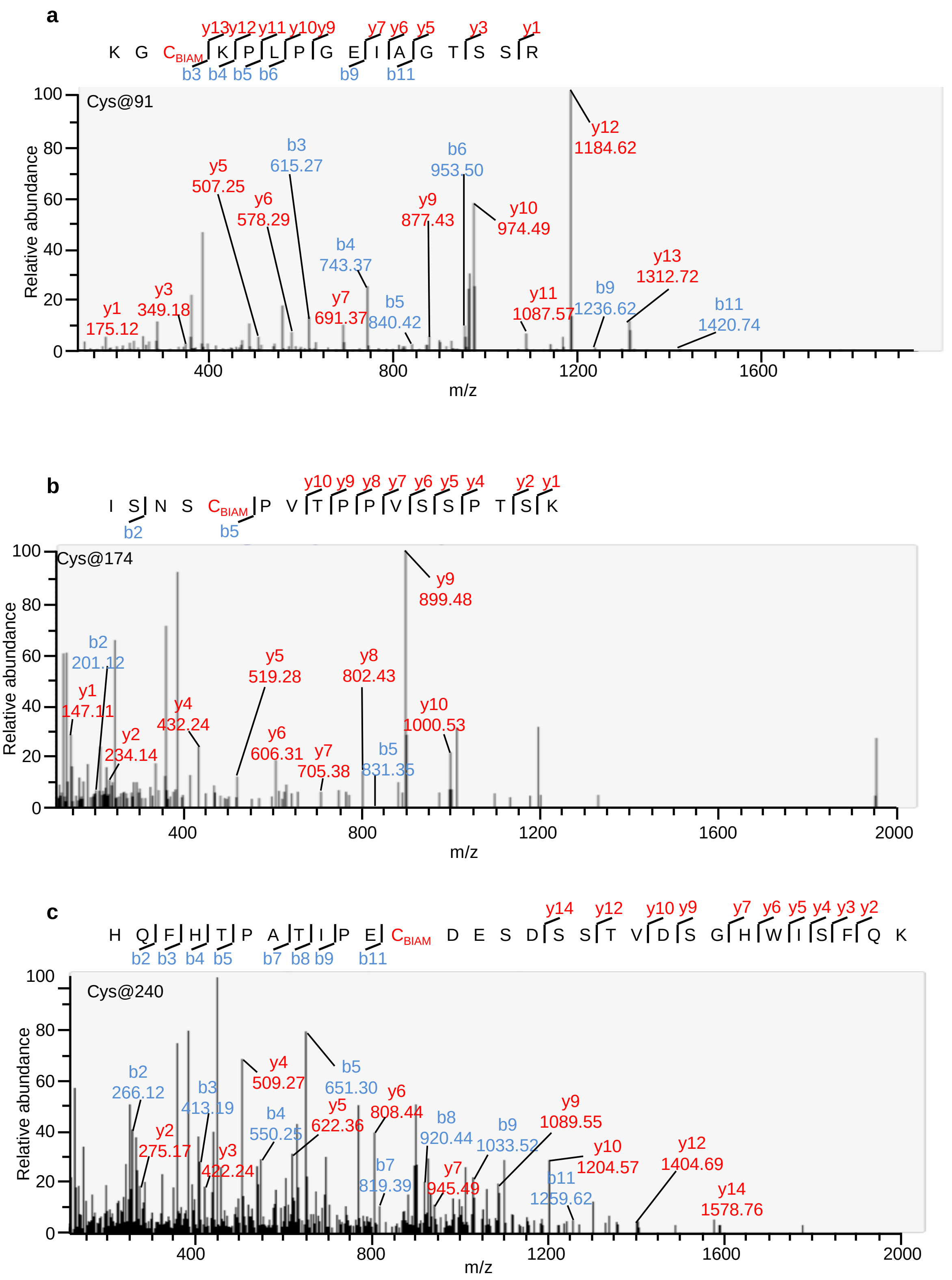

Supplementary Figure 5. Mass spectrometry analysis of the tryptic fragments of MBP-BZR1 protein sequentially treated with H_2O_2, NEM, and BIAM.

(a-c) Cys-91 (a), cys-174 (b) and cys-240 (c) charged with BIAM was identified as an H_2O_2-sensitive residue.

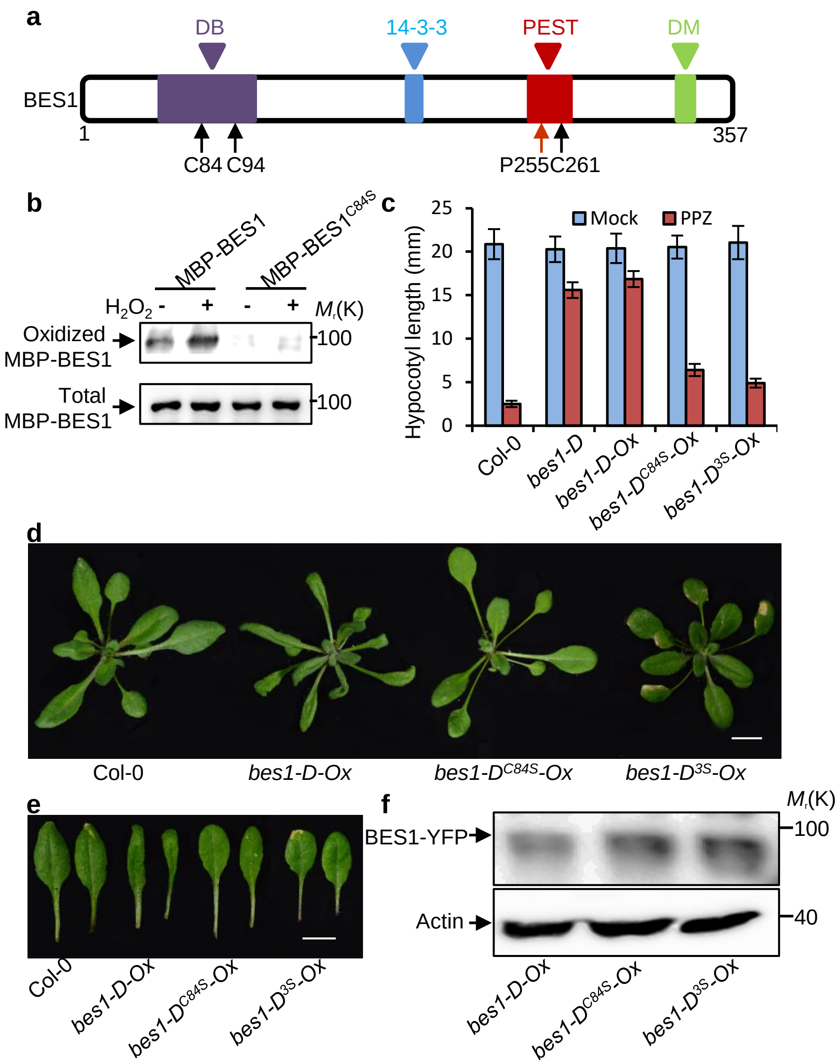

Supplementary Figure 6. Cys-84 is required for the function of BES1.

(a) Illustration depicted BES1 protein structure. Purple box indicates DNA binding domain; dark blue indicates 14-3-3 binding domain; dark red indicates proline, glutamic acid, serine, and threonine rich (PEST) domain; light green indicates BIN2 binding domain (DM); the black arrows refer to the cysteine residues; and the red arrow refers the 255th proline. **(b)** Identification of the H_2O_2-sensitive cysteine residues in MBP-BES1 using the biotin-switch method. MBP-BES1 and MBP-BES1^{C84S} proteins pretreated with H_2O_2 were firstly incubated with NEM to irreversibly block the free thiols of MBP-BES1. The NEM-modified MBP-BES1 and MBP-BES1^{C84S} proteins were treated with DTT to reduce the pre-existing oxidized cysteine in proteins. The newly exposed free thiol groups were then relabeled with BIAM. The BIAM-tagged proteins in the samples were then immunoprecipitated with streptavidin beads and detected by western blot with anti-MBP antibody. **(c)** The bes1-D^{C84S} and bes1-D$^{C84, 94, 261S}$(bes1-D^{3S}) mutation attenuated the PPZ-resistant phenotype of *bes1-D*. Seedlings were grown in the dark on ½ MS medium with or without 2 μM PPZ for six days, and the hypocotyl length of each seedling were measured. Error bars indicated s.d. (n=30 plants). **(d,e)** Phenotype of mutated BES1 transgenic plants (d) and leaves (e) grown in soil for 3 weeks under long-day condition. Scale bar, 10 mm. **(f)** Immunoblot analyzed the protein levels of BES1 in different transgenic plants by probing with anti-YFP antibody. An antibody against actin was used to verify equal protein loadings.

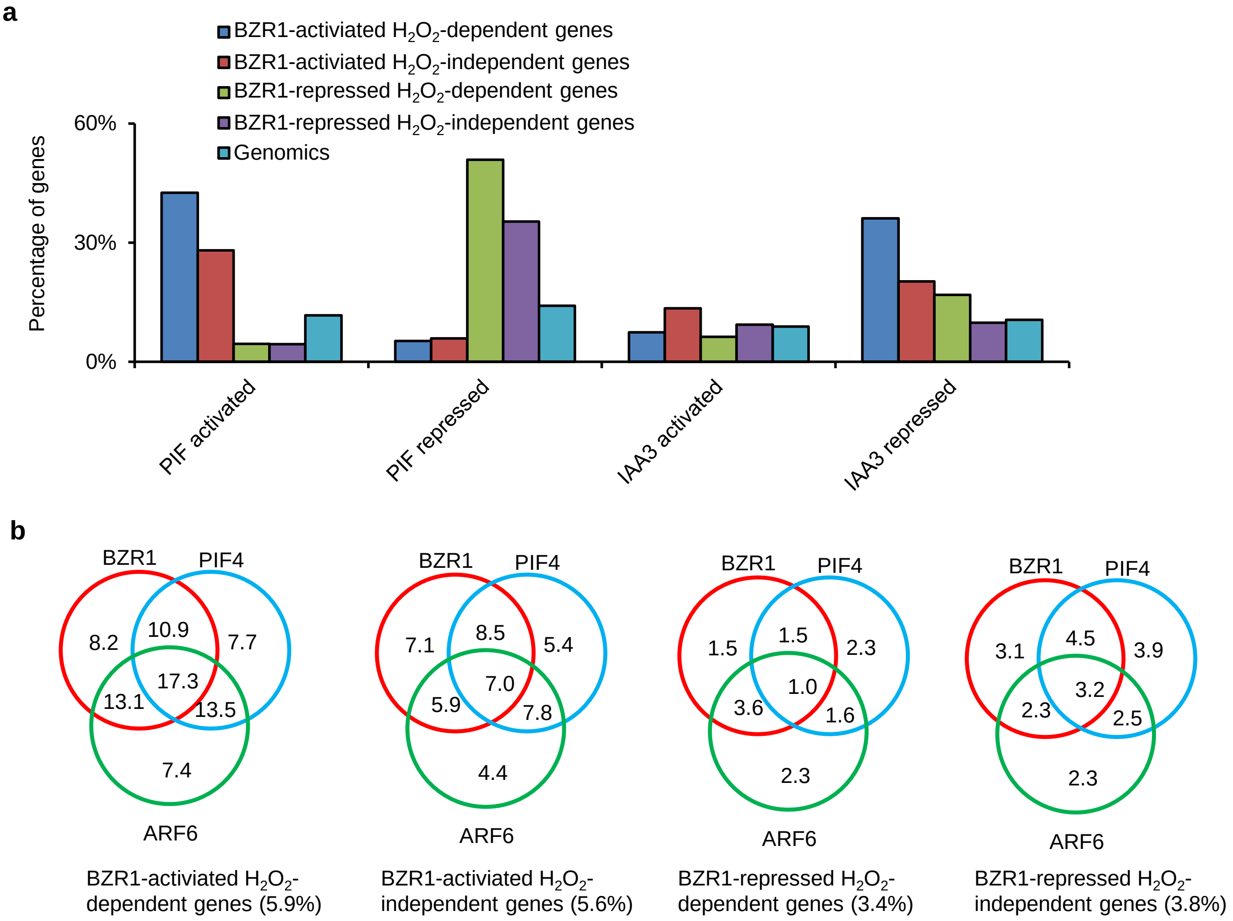

Supplementary Figure 7. H_2O_2-dependent BZR1-activated genes are enriched in the genes directly regulated by PIF4 or ARF6.

(a) Frequency of shown BZR1-regulated H_2O_2-dependent or H_2O_2-independent genes in the PIF- or IAA3- regulated genes. **(b)** Venn diagram shows that H_2O_2-dependent BZR1-activated genes are enriched in the common binding targets of BZR1, PIF4 and ARF6. Numbers in the Venn diagram indicate percentage of corresponding genes (e.g., BZR1-activated H_2O_2-dependent genes) in each section. Numbers in parentheses indicate percentage of genes in total Arabidopsis genome.

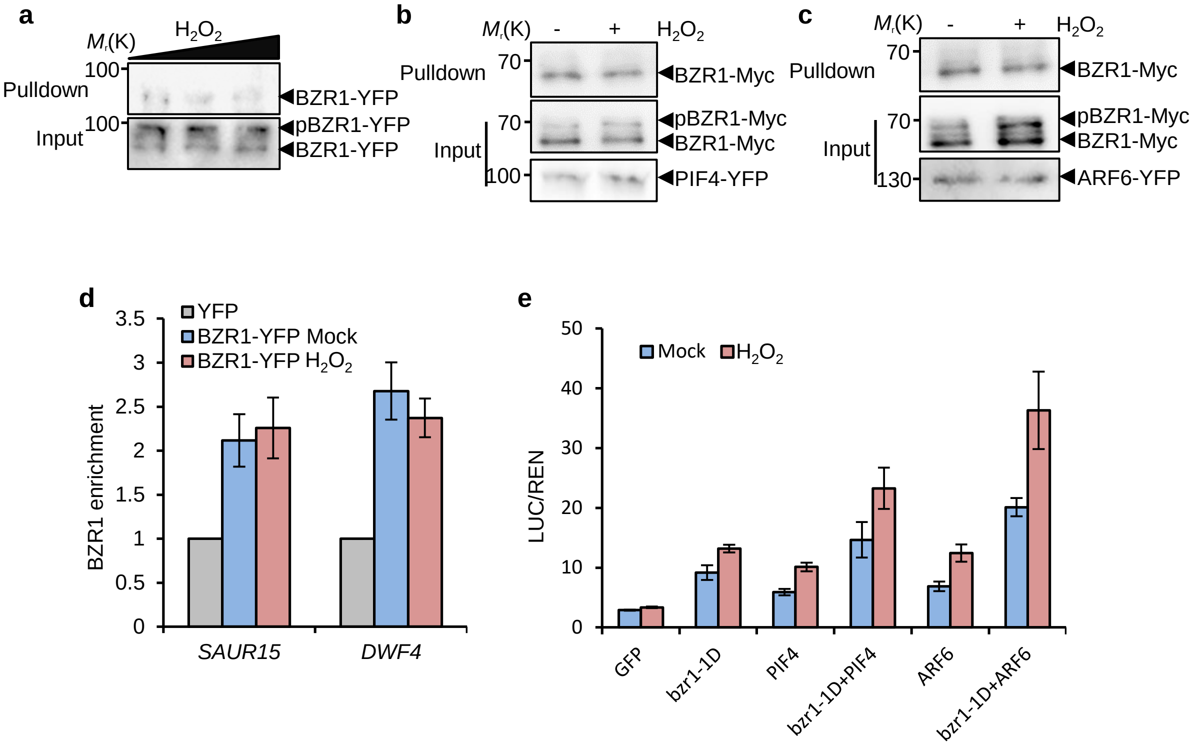

Supplementary Figure 8. H_2O_2 has no significant effects on the DNA binding ability of BZR1.

(a) DNA-protein pull-down assays was performed with biotinylated DNA fragment from the *SAUR15* promoter and lysates of *p35S:BZR1-YFP* plants that were pretreated with different concentrations of H_2O_2 for 30 mins. The DNA-bound proteins were immunoblotted using anti-YFP antibody. **(b,c)** The Arabidopsis mesophyll protoplasts expressing BZR1-Myc and PIF4-YFP (b), or BZR1-Myc and ARF6-YFP (c), were treated with or without 100 μM H_2O_2 for 30 mins, and then harvested to perform immunoprecipitation with streptavidin beads containing biotinylated DNA fragment from the *SAUR15* promoter. Target proteins were detected in western blots using anti-Myc and anti-YFP antibodies. **(d)** H_2O_2 had no significant effect on the levels of BZR1-DNA binding *in vivo*. ChIP was performed using GFP-trap agarose beads followed by qPCR analysis. The level of BZR1 binding was calculated as the ratio between *p35S:BZR1-YFP* and the *p35S:YFP* control, normalized to that of the control gene *PP2A*. Error bars, s.d. of three biological repeats. **(e)** Transient reporter gene assays showed H_2O_2 increased the transcription activity of BZR1. Arabidopsis mesophyll protoplasts were transformed with the dual luciferase reporter construct containing *pPRE5:LUC* (luciferase) and p*35S:REN* (renilla luciferase), and constructs overexpressing the indicated effectors. Before harvesting, the protoplasts were treated with or without 100 μM H_2O_2 for 30 mins. The LUC activity was normalized to REN. Error bars, s.d. of three biological repeats.

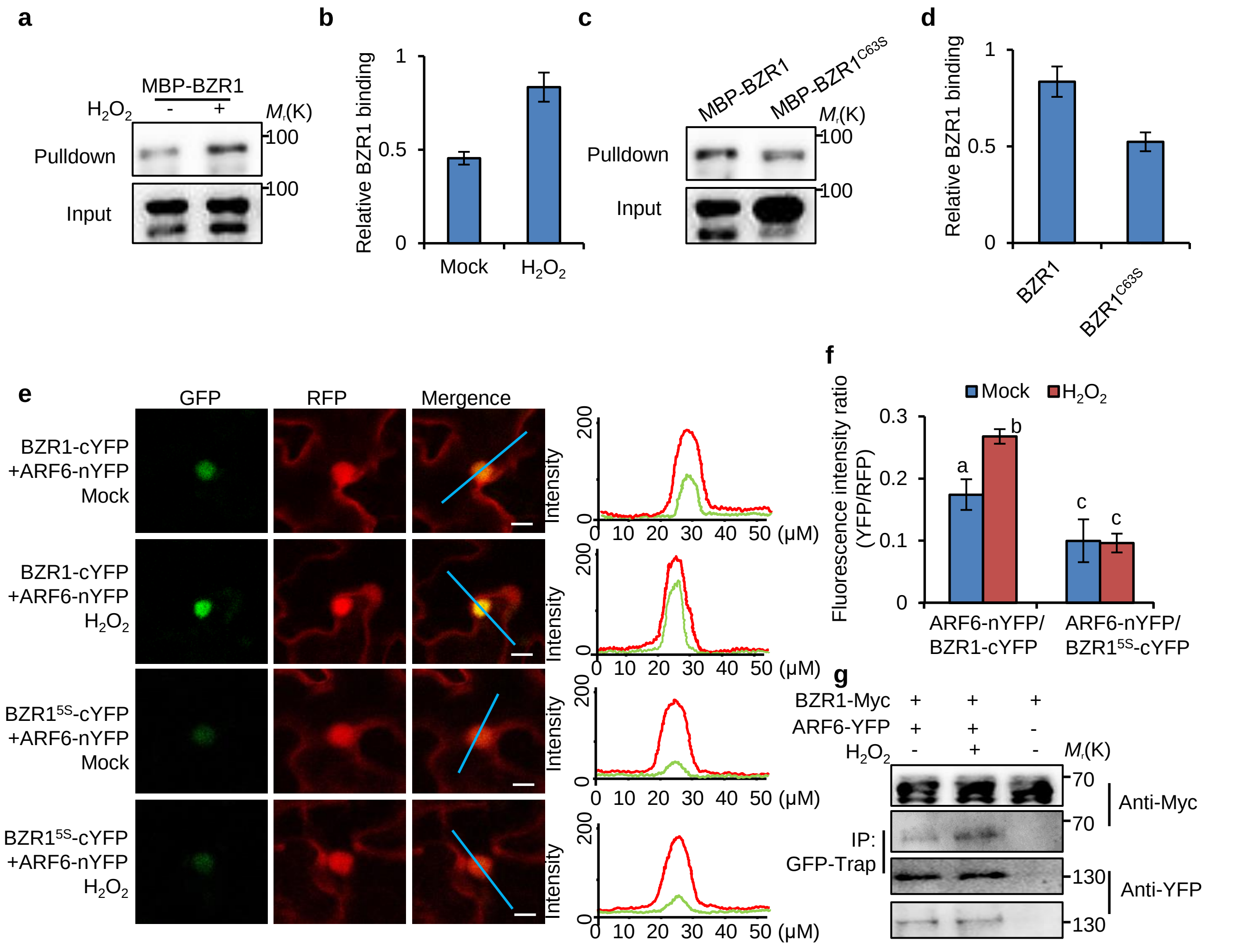

Supplementary Figure 9. H_2O_2 enhances the interaction of BZR1 and ARF6 *in vivo* and *in vitro*.

(a-d) Pull-down assays showed that H_2O_2 (a,b) enhanced, while Cys-63 mutation to Ser (c,d) reduced the BZR1-binding ability to ARF6. BZR1, and BZR1^{C63S} fused with MBP tags were pulled down by GST-ARF6 and immunoblotted using anti-MBP antibody. Panel (b) and (d) show the quantification of pull-down assays in the panel (a) and (c), respectively. The column indicated the ratio of the mean gray value of immunoblot bands between pull-down and input. Error bars represented the s.d. of three independent experiments. *$p < 0.05$, as determined by a Student's *t* test. **(e,f)** RBiFC confocal images show that H_2O_2 enhances, while mutation of cysteine reduces the BZR1-binding affinity to ARF6 in plants. The fluorescent signal of YFP and RFP were taken using LSM700 microscope from Zeiss. The fluorescent intensities were determined along a line drawn on the confocol images using ImageJ software. Scale bar, 10 μm. Error bars, s.d. (n=50 images). Different letters above the bars indicated statistically significant differences between the samples (two-way ANOVA, $p < 0.05$). **(g)** CoIP assays showed that H_2O_2 increased the interaction between BZR1 and ARF6 in plants. The Arabidopsis mesophyll protoplast transformed with BZR1-Myc and/or ARF6-YFP were treated with or without 100 μM H_2O_2 for 1 hr, and then harvested to perform immunoprecipitation using GFP-Trap agarose beads. Target proteins were detected in western blots using anti-Myc and anti-YFP antibodies.

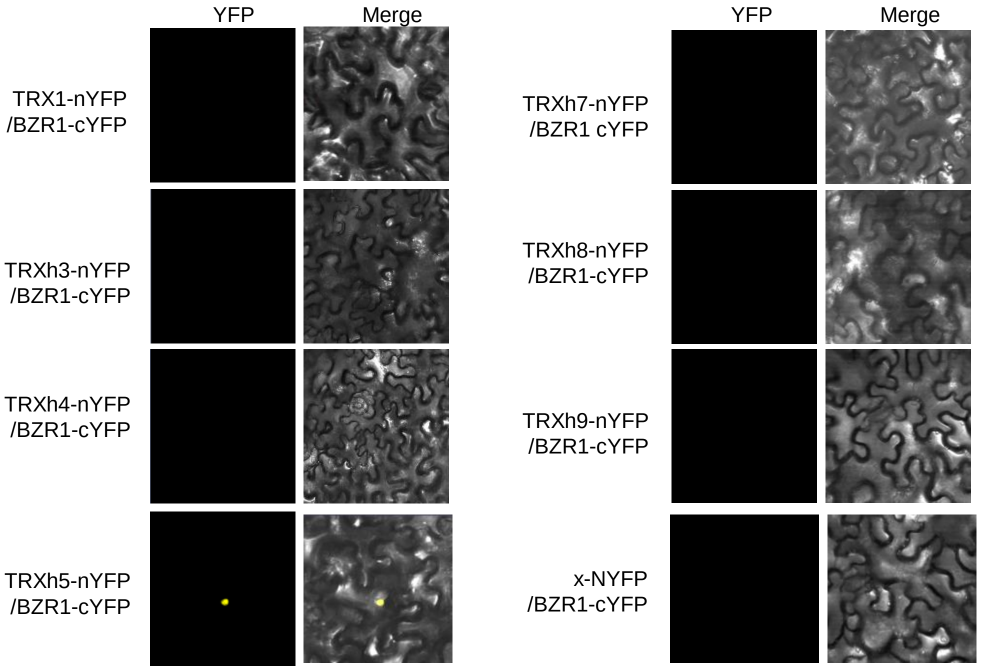

Supplementary Figure 10. TRXh5 specifically interacts with BZR1 in tobacco leaves.

Bimolecular fluorescence complementation (BiFC) assays analyzed the interaction between BZR1 and members of TRX-h family in tobacco leaves. Leaf epidermal cells of *N. benthamiana* were cotransformated with BZR1-cYFP and members of TRX-h proteins fused to the amino-terminal half of YFP (TRXhs-nYFP). The fluorescent signals were taken using LSM700 microscope from Zeiss. Merge, overlay of the YFP and light images.

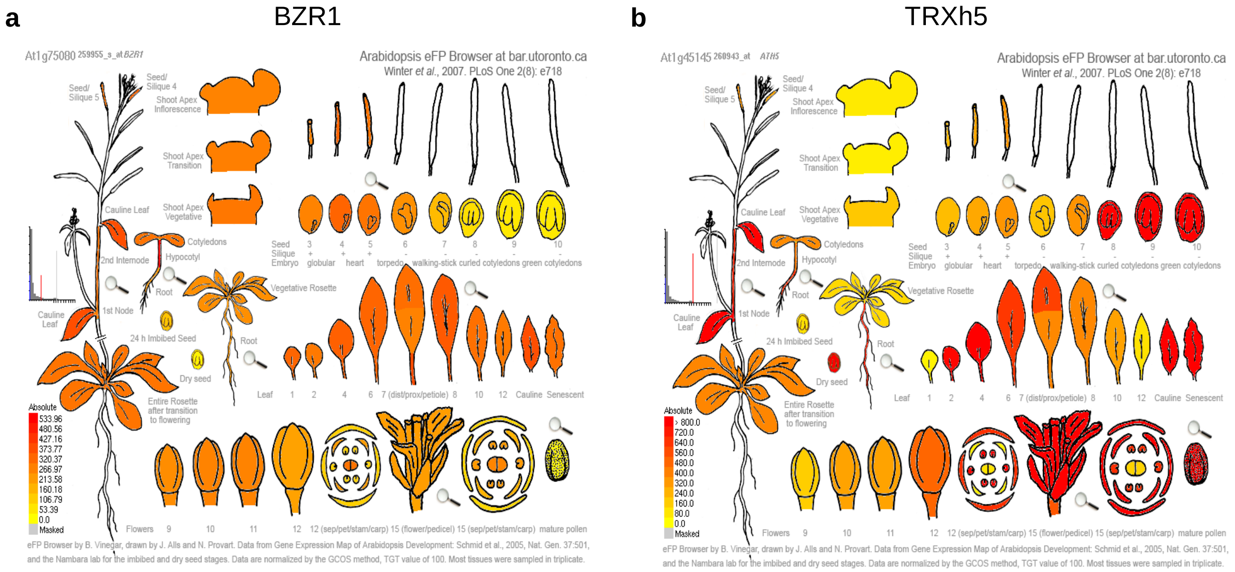

Supplementary Figure 11. Overlapping expression pattern of *BZR1* and *TRXh5* in plants.

(a,b) The expression pattern of *BZR1* (a) and *TRXh5* (b) were generated using the Arabidopsis eFP browser at *http://bbc.botany.utoronto.ca/efp/cgi-bin/efpWeb.cgi* (Winter et al., 2007). The color scale at the left bottom corner in each panel indicates the absolute expression levels of each individual gene: red means higher while yellow indicates lower expression levels.

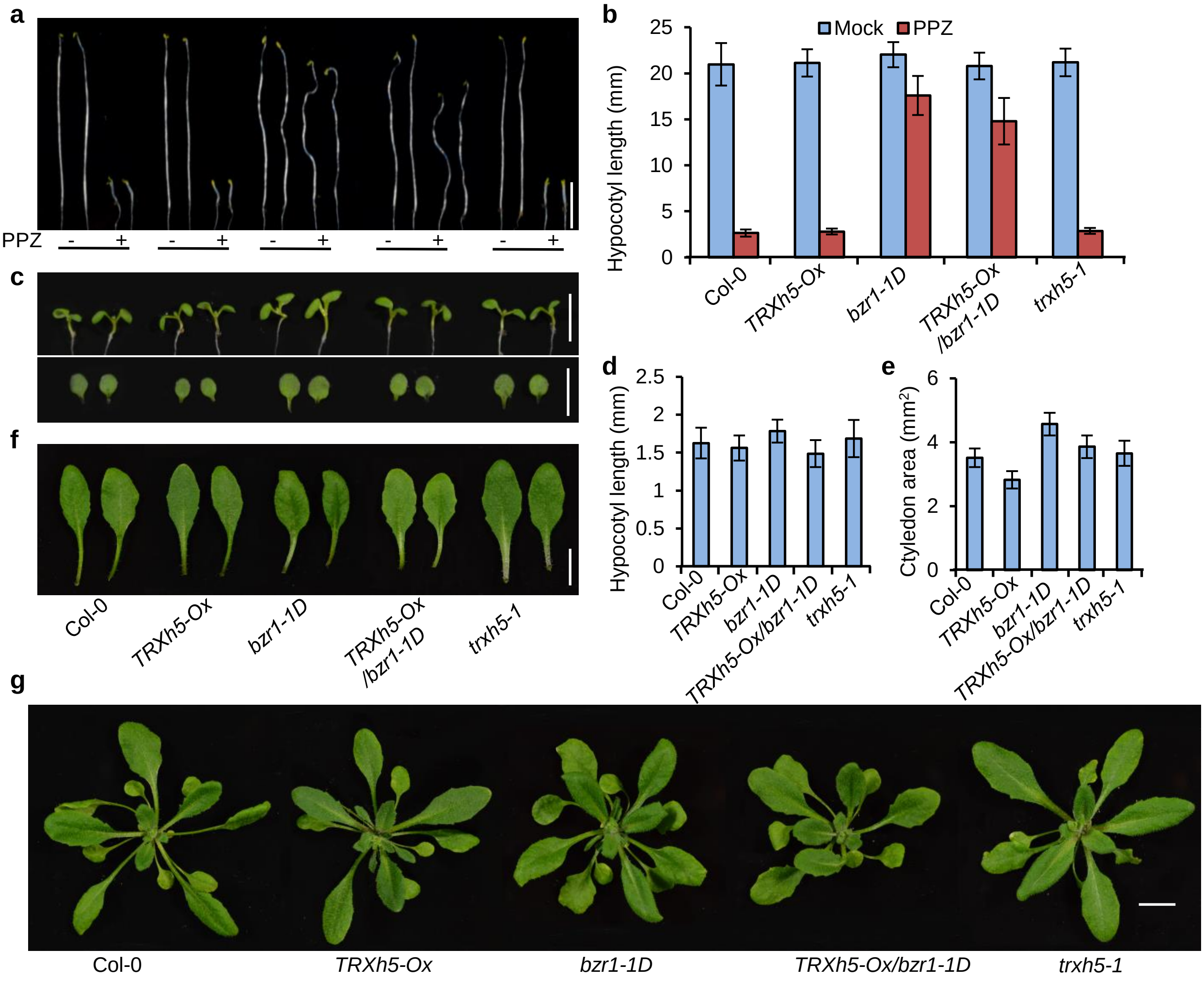

Supplementary Figure 12. Overexpression of *TRXh5* partial suppresses the function of BZR1 on cell elongation.

(a,b) *TRXh5* overexpression attenuated the resistance of *bzr1-1D* to PPZ. Seedlings were grown on ½ MS medium containing 2 μM PPZ in the dark for six days, the hypocotyl length of each seedling were measured. Scale bar, 5 mm. Error bars indicated s.d. (n=30 plants). **(c-e)** Overexpression of *TRXh5* suppressed the large cotyledons phenotype of *bzr1-1D*. Wild type Col-0, *TRXh5-Ox, bzr1-1D, TRXh5-Ox/bzr1-1D and trxh5-1* were grown on ½ MS medium under constant light for 7 days. The hypocotyl length and cotyledon area of each seedling were measured. Scale bar, 5 mm. Error bars indicated s.d. (n=30 plants). **(f,g)** Phenotype of Col-0, *TRXh5-Ox*, *bzr1-1D*, *TRXh5-Ox/bzr1-1D* and *trxh5-1* plants grown in soil under long-day condition for 3 weeks. Scale bar, 10 mm.

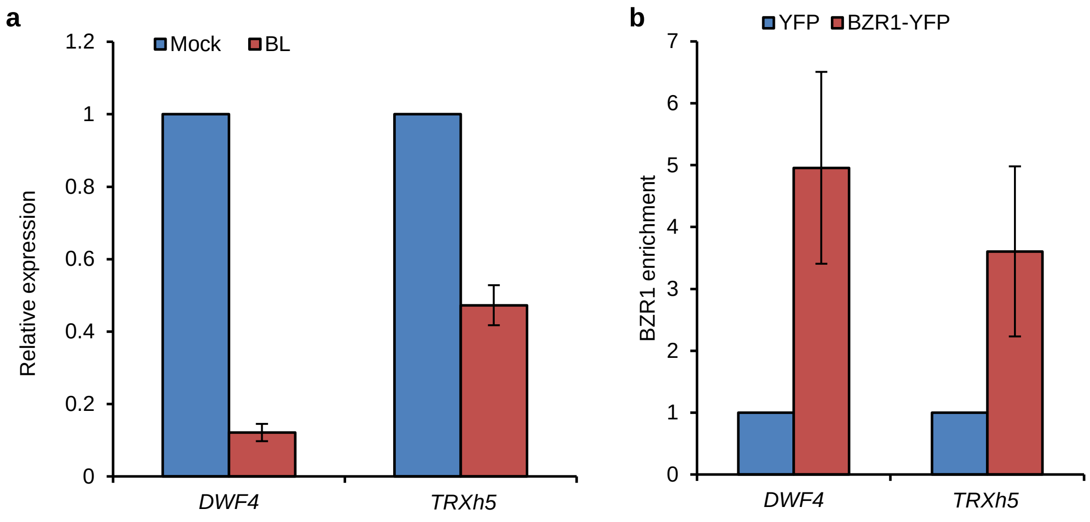

Supplementary Figure 13. BZR1 directly binds and represses the expression of *TRXh5*.

(a) BR repressed the expression of *TRXh5* and *DWF4*. *PP2A* was used as the internal control. Error bars indicate standard deviation from three biological repeats. **(b)** Quantitative ChIP-PCR analysis of BZR1 binding to the promoter of *TRXh5* and *DWF4*. The chromatin of *pBZR1:BZR1-YFP* and *p35S:YFP* transgenic plants was immunoprecipitated with GFP-trap, and the precipitated DNA was quantified by quantitative PCR. Enrichment of DNA was calculated as the ratio between *pBZR1:BZR1-YFP* and *p35S:YFP*, normalized to that of the *PP2A* coding region. Error bars indicate standard deviation of three biological repeats.

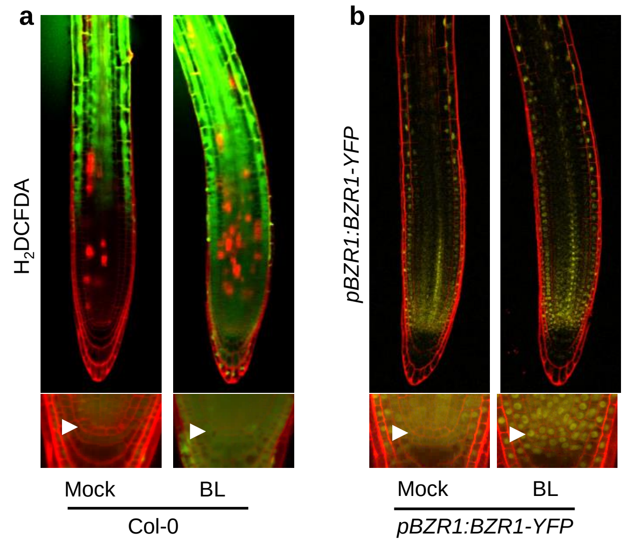

Supplementary Figure 14. The similar distribution patterns of H_2O_2 and activated-BZR1 in root tips.

(a) H_2DCFDA staining for H_2O_2 in the primary root tips of Col-0 treated with or without BR. **(b)** Confocal images of BZR1-YFP localization in a root tip of the *pBZR1:BZR1-YFP* plant with or without BR treatment. Seedlings were grown on ½ MS medium for 4 days, and then transferred to ½ MS liquid medium containing mock solution or 10 nM BL for 30 mins. Arrowheads indicate QC cell layers. Roots were stained with propidium iodide (red). Fluorescent signals were taken using LSM700 microscope from Zeiss.

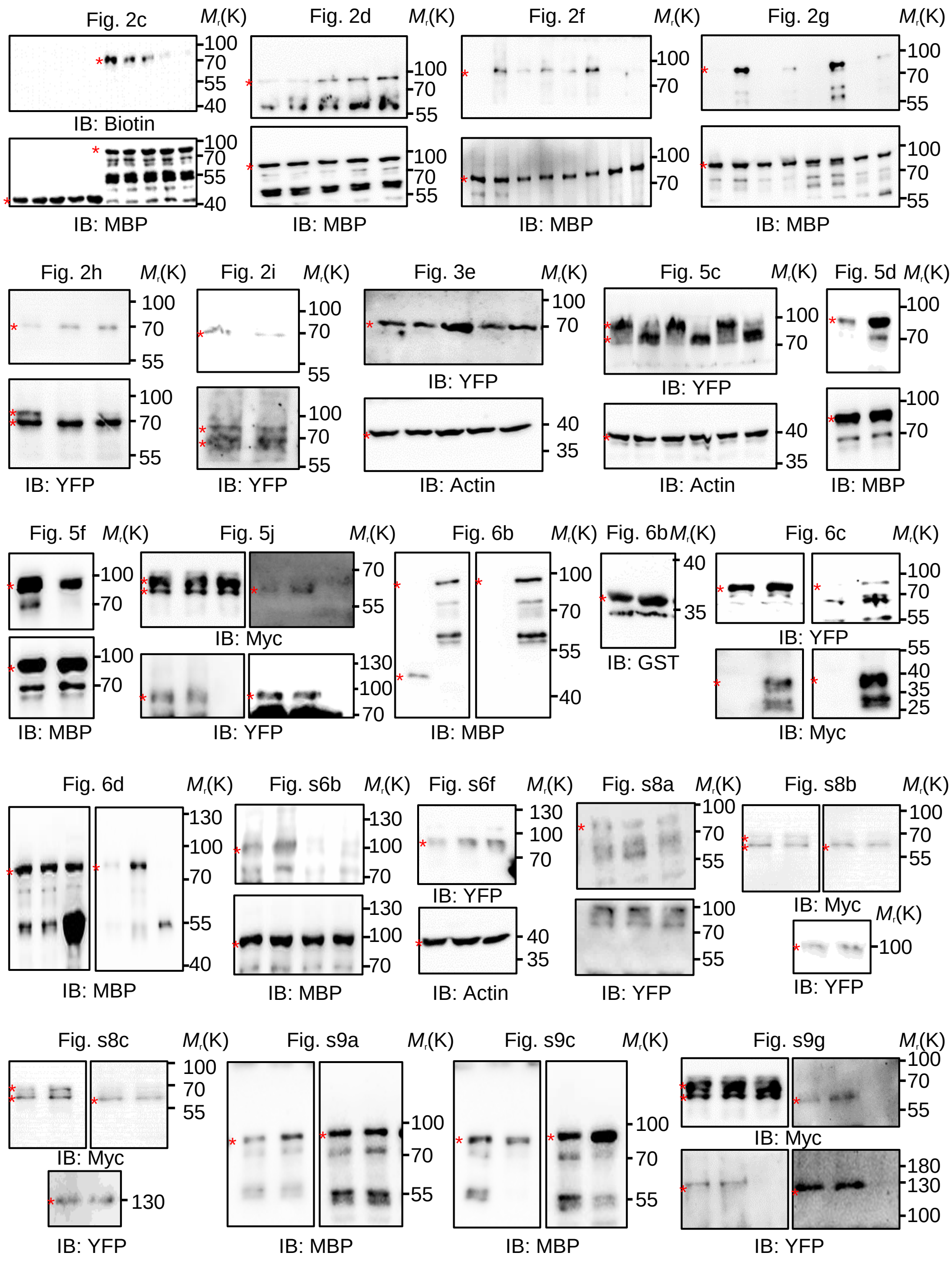

Supplementary Figure 15. Full scan data of immunoblots. Red asterisks indicate the correct bands shown in the figures.
