# Supplementary material for: Hydrogen peroxide positively regulates brassinosteroid signaling through oxidation of the BRASSINAZOLE-RESISTANT1 transcription factor
Source: Nat Commun. 2018 Mar 14;9:1063. doi: 10.1038/s41467-018-03463-x (PMC5852159; doi:10.1038/s41467-018-03463-x)
Supplement: Supplementary file 3 — Description of Additional Supplementary Files [file 41467_2018_3463_MOESM3_ESM.pdf]

Description of Supplementary Files

File Name: Supplementary Data 1
Description: **BZR1-regulated genes in mock condition.** Col-0 and *bzr1-1D* were grown on the medium containing 2 µM PPZ for 5 days in the dark for RNA-Seq analysis. Differentially expressed genes between *bzr1-1D* and Col-0 were defined by a 1.5-fold expression difference with a possibility >0.8.

File Name: Supplementary Data 2
Description: **BZR1-regulated genes in DPI condition.** Col-0 and *bzr1-1D* were grown on the medium containing 2 µM PPZ and 1 µM DPI for 5 days in the dark for RNA-Seq analysis. Differentially expressed genes between *bzr1-1D* and Col-0 were defined by a 1.5-fold expression difference with a possibility >0.8.

File Name: Supplementary Data 3
Description: **BZR1-regulated genes in DPI and H_2O_2 condition.** Col-0 and *bzr1-1D* were grown on the medium containing 2 µM PPZ, 1 µM DPI and 0.3mM H_2O_2 for 5 days in the dark for RNA-Seq analysis. Differentially expressed genes between *bzr1-1D* and Col-0 were defined by a 1.5-fold expression difference with a possibility >0.8.

File Name: Supplementary Data 4
Description: **DPI affected genes in *bzr1-1D* background.** Seedlings of *bzr1-1D* were grown on the medium containing 2 µM PPZ and/or 1 µM DPI for 5 days in the dark for RNA-Seq analysis. Differentially expressed genes between DPI treatment and no treatment were defined by a 1.5-fold expression difference with a possibility >0.8.

File Name: Supplementary Data 5
Description: **Oligonucleotide sequences used in this paper.**
